# Editorial: Women in science - nuclear medicine 2024

**DOI:** 10.3389/fmed.2025.1684663

**Published:** 2025-10-09

**Authors:** Bianca Gutfilen, Marigdalia K. Ramirez-Fort

**Affiliations:** ^1^Department of Radiology, School of Medicine, Universidade Federal do Rio de Janeiro, Rio de Janeiro, Brazil; ^2^Department of Biodefense, BioFort Corp., Guaynabo, Puerto Rico; ^3^Department of Nuclear Medicine, Veterans Affairs Greater Los Angeles Healthcare System, Veterans Health Administration, Los Angeles, CA, United States

**Keywords:** women, Nuclear Medicine, Radiation Sciences, gender equity, prostate-specific membrane antigen (PSMA), folate hydrolase-1 (FOLH1), HER2/neu, Alzheimer's disease

Women are underrepresented in science, particularly at the graduate, postgraduate and faculty levels (1). In 2022, women accounted for only 31% of researchers worldwide; the only regions approaching gender parity were in Latin America and in the Caribbean (2). Ross et al. further demonstrated that women are systematically undercredited and marginalized for their scientific aptitude and contributions compared to men (3). Women are less likely than their male colleagues to be named as authors on scientific papers or to receive credit as named inventors on inventions or patents. Qualitative data (e.g., interviews, surveys, etc.) further support the misrepresentation of women's contribution to science and medicine (3).

A known example of this misrepresentation is the story of Rosalind Franklin, who took the first radiographic photographs of DNA, confirming that DNA had two helices. Franklin elucidated the basis of James Watson and Francis Crick's discovery that DNA is a double-helical polymer, but was never credited for her role in the discovery of the DNA structure. Another example of the gender disparity described by Ross et al. was recently documented in the United States Federal Court for the District of Puerto Rico (Case No. 3:19-cv-01631-FAB). As a resident physician training in a Radiation Oncology residency program, a woman physician-scientist was subjectively labeled as academically deficient compared to her male colleagues. Soon thereafter, she informed her residency supervisor that she had a manuscript accepted for publication on the then novel topic of prostate specific membrane antigen [PSMA; folate hydrolase-1 (FOLH1)], as the first author in the International Journal of Radiation Oncology, Biology and Physics (4). The residency competency committee then labeled her as having delusions of grandeur, presumably because they were incredulous that a woman resident could accomplish this. The Radiation Oncology faculty then removed her from the residency training program, despite objective measures of academic competency (5–7).

Maria Skłodowska-Curie made it clear that women can play a crucial role in the development and advancement of Radiation Sciences, particularly Nuclear Medicine. In fact, several women have “paved the way” in pioneering Radiation Science and Nuclear Medicine. Edith Quimby elucidated how to optimize the radiotherapeutic index of radiopharmaceuticals. Irene Joliot-Curie co-discovered that radioactive elements could be artificially produced from stable elements. Rosalyn Sussman Yalow developed the radioimmunoassay technique. Chien-Shiung Wu, disproved “the law of conservation of parity” by discovering that cobalt-60 particle emission is asymmetrical. Tikvah Alper identified the infectious agent in Scrapie; by irradiating scrapie samples with different wavelengths of UV light, Alper was able to prove that the infectious agent could replicate despite having no nucleic acid. In 2020, a mother-daughter research team published their inventive methods for cellular-level dosimetry of radiopharmaceuticals (8, 10, 11).

It is essential to acknowledge that women in Nuclear Medicine encounter gender-based challenges and obstacles in the battle to be recognized for their intellectual contributions. Further, the women that encounter these gender-based challenges and obstacles may suffer tangible career, financial and reputational damages. Nevertheless, women consistently demonstrate competence, dedication, and excellence in their professional practices. This Research Topic aimed to mitigate these damages by creating opportunities to recognize and honor the recent contributions of women in Nuclear Medicine while also promoting gender equality.

Hu et al. developed ^131^I-labeled HER2 affibodies as targeted radionuclide therapy (TRNT) agents for HER2-positive ovarian carcinoma. The YZ_HER2:v2_ affibody targeting HER2 was synthesized through genetic recombination. Radiopharmaceutical tumor accumulation, retention, and advantageous distribution were observed in mice with HER2-positive tumors. Mice treated with ^131^I-YZ_HER2:v2_ showed reduced tumor growth and prolonged survival (Hu et al.). Sharing information on techniques for developing novel radiopharmaceuticals that target a variety of known proteins expressed by cancers is essential to optimizing radiation dose delivery to disseminated cancers.

In an Opinion article, Ramirez-Fort et al. drew attention to the fact that the use of gender- and disease-exclusive language in science and medicine can lead to the exclusion of patients, particularly genotypic women, from important diagnostic and therapeutic opportunities. One example is the term “prostate-specific membrane antigen”, which is widely used in oncology. The PSMA protein, which is encoded by the *FOLH1* gene, is present in all human solid tumors. Maintaining the term “PSMA” perpetuates a gendered view and a false exclusive association with prostate cancer. On 28 March 2025, the United States Food and Federal Drug Administration (FDA) expanded its indication for a “PSMA”-targeted radiopharmaceutical to include the treatment of metastatic prostate cancer only. The FDA's approval of this misnamed radiopharmaceutical forms the basis for Centers for Medicare and Medicaid reimbursement. Since it has been known for decades that “PSMA” is expressed within the lumen of all cancer neovessels, the authors argue that standardizing the protein's name from PSMA to FOLH1 is the first important step toward achieving gender equality in the use of PSMA-targeted radiopharmaceuticals in all solid tumors such as breast and ovarian cancers (Ramirez-Fort et al.) (9).

With a focus on future clinical applications, Burgard et al. aimed to identify suitable prognostic dynamic parameters derived from baseline and follow-up [18F] FDG PET/CT and dual-tracer imaging PET/CT, for monitoring metastatic castration-resistant prostate cancer (mCRPC) patients who are not responding to PSMA-targeted therapy. The researchers identified a novel biomarker called “change of glucometabolic activity per PSMA expression for total lesions” (cGAPTL) which is a normalized ratio of whole-body lesion glycolysis to whole-body lesion PSMA expression. Importantly, cGAPTL is shown to reliably predict overall survival in mCRPC patients who do not respond to [177Lu] Lu-PSMA-617 radioligand therapy (Burgard et al.). It will be exciting to see how cGAPTL can aid in optimizing radiopharmaceutical treatment planning for disseminated prostate cancer.

In their Original Research article, Peretti et al., described the prevalence of PET and MRI-quantified ATN profiles in two separate patient cohorts to determine whether neuroimaging-based ATN profiles are standardizable. Currently, Alzheimer's disease is staged by the level of neurodegeneration (N) and is histopathologically diagnosed based on amyloid (A) plaques and neurofibrillary tau (T) tangles. Together, these parameters form the ATN classification system, which has the potential to be used as a standardized method for evaluating disease progression within clinical trials or the efficacy of new drugs. This study shows that neuroimaging alone establishes reproducible and consistent ATN profiling among centers with different clinical cohorts and imaging protocols (Peretti et al.).

The editors of this Research Topic conclude that the authors of this Research Topic on Women in Science have made important intellectual contributions to Nuclear Medicine. We recommend that the scientific and medical community continue to bring awareness to the existence of and the far-reaching damages associated with gender inequality.
